# Long-time behavior of swimming *Euglena gracilis* in a heterogenous light environment

**DOI:** 10.3389/fcell.2023.1133028

**Published:** 2023-02-20

**Authors:** Kazuki Muku, Hiroshi Yamashita, Touya Kamikubo, Nobuhiko J. Suematsu, Makoto Iima

**Affiliations:** ^1^ Department of Integrated Arts and Sciences, Hiroshima University, Higashihiroshima, Japan; ^2^ Graduate School of Integrated Sciences for Life, Hiroshima University, Higashihiroshima, Japan; ^3^ Department of Mathematics, Hiroshima University, Higashihiroshima, Japan; ^4^ Meiji Institute for Advanced Study of Mathematical Sciences(MIMS), Meiji University, Nakano, Japan; ^5^ Graduate School of Advanced Mathematical Sciences, Meiji University, Nakano, Japan

**Keywords:** E. gracilis, diorama environment, heterogeneous light condition, long time scale orbit, curvature radius

## Abstract

The cell motion of *Euglena gracilis* in homogeneous and heterogeneous light environments was analyzed. Homogeneous and heterogeneous environments were prepared, with only a red color or with a red circle surrounded by brighter white regions, respectively. In a heterogeneous environment, the cells move into the red circle. Swimming orbits at 1/25 s intervals for 120 s were analyzed. The speed distribution of the 1 s-averaged cell orbits in a homogeneous environment was different from that in a heterogeneous environment, where the faster swimming fraction was enhanced. The relationship between speed and curvature radius was analyzed using a joint histogram. Histograms for short timescale motion, constructed by 1 s-averaged orbits, suggest that the cell swimming curves are not biased, while those for long timescale motion, constructed by 10 s-averaged orbits, suggest that the cell swimming curves are biased in the clockwise direction. Furthermore, the curvature radius determines the speed, which does not seem to depend on the light environment. The mean squared displacement in a heterogeneous environment is larger than that in a homogeneous environment on a 1 s timescale. These results will be the basis for constructing a model for the long-time behavior of photomovement for light differences.

## 1 Introduction

Many microorganisms swim using cilia and flagella or move by pseudopods to improved habitats, boosting their survival chances. Such motion is due to information processing by sensing external signals. Cell movements to their preferred surroundings are called *taxis*, including chemotaxis (chemical concentration), gravitaxis (gravity), and phototaxis (light intensity). Here, we focused on the photomovement of *Euglena gracilis* under heterogeneous light conditions.

*E. gracilis*, a model microorganism with a body length of 50 *μ*m, swims using a single long flagellum outside the body. The paraflagellar body (PFB), a light-sensing organ of *E. gracilis*, senses light through a chemical, photo-activated adenyl cyclase (PAC), specifically by a step-up photophobic response (Iseki et al., 2002). The light-sensing system functions when *E. gracilis* swims by rotating its body along the long axis, causing the stigma (red spot) on the PFB to change light intensity to perceive the light direction (Diehn, 1973; Kato and Shinomura, 2019). Because *E. gracilis* has a single PFB, it must process light information at a single point in the PFB.

In a heterogeneous environment, *E. gracilis* swims to a location with a specific light intensity (Giometto et al., 2015; Ogawa et al., 2016). Consequently, they require a method of information processing to detect heterogeneous light distribution.

To date, three types of photomovements of *E. gracilis* are known (Diehn, 1973): (a) phototaxis: swimming direction changes according to the light vector; (b) photokinesis: swimming speed dependency on the light intensity; and (c) photophobic reponse: cell motion changes due to the change in light intensity.

Photophobic response is a photomovement related to cell behavior in an environment with spatial light distribution. The action spectrum, or the change in motion caused by a change in light intensity at a specific wavelength, has been studied. Because the action spectrum for the abrupt increasing light intensity correlates well with the absorption spectra of PAC, PAC is thought to be related to a step-up photophobic response (Iseki et al., 2002). Recently, a polygonal cell orbit with a sudden increase in light intensity has been reported (Tsang et al., 2018). This behavior is suggested to sense the spatial light distribution with a similar ‘run-and-tumble’ mechanism of bacterial chemotaxis (e.g., *Escherichia coli*) (Berg, 1993; Tsang et al., 2018).

It is unclear whether this mechanism is valid in a more general light environment, thus cell motion in a heterogeneous environment should be analyzed in detail. We focused on the long-term behavior of cell motion, which has not been studied as intensively as individual swimming (Rossi et al., 2017; Giuliani et al., 2021) or euglenoid motion (Noselli et al., 2019). When the randomness of the cell motion is focused on, the mean squared displacement (MSD) can be an indicator. For an active Brownian particle, which has a constant speed with the direction defined by Brownian motion (Marchetti et al., 2016), the exponent depends on the timescale; the behavior is ballistic (MSD ∼ *t* ^2^) for shorter times than the characteristic timescale, called persistence, and diffusive for longer times (MSD ∼ *t* ^1^). A similar transition has been reported for the orbit due to the ‘run-and-tumble’ motion observed in *E. coli* (Solon et al., 2015) and the Lévy walk model (Zaburdaev et al., 2015).

For *E. gracilis* motion, the exponent of MSD has been reported 1.89 (Ogawa et al., 2017); the cell motion after a sudden change in light intensity gives various exponents for shorter timescales of less than 10 s (Tsang et al., 2018), suggesting that the cell motion behavior depends on the spatial or temporal light change; however, the relationship between the exponent and the orbit in physical space is not fully understood. Particularly, *E. gracilis* cell motion adapted to heterogeneous light intensities has not been studied. Because there are several timescales for adaptation to environmental change (Ozasa et al., 2019), we expect that swimming behavior will change with the heterogeneous environment.

Here, we investigated the motion of *E. gracilis* in a spatially heterogeneous but stationary manner. We prepared homogeneous and heterogeneous environments and smoothed orbits to eliminate short-time helical motions owing to swimming details. The speed histograms for these environments were different. The orbits’ shape was characterized by the curvature radius and the local speed; their functional relationships were determined. Additionally, MSD exponents in these environments differed.

## 2 Materials and methods

**FIGURE 1:**
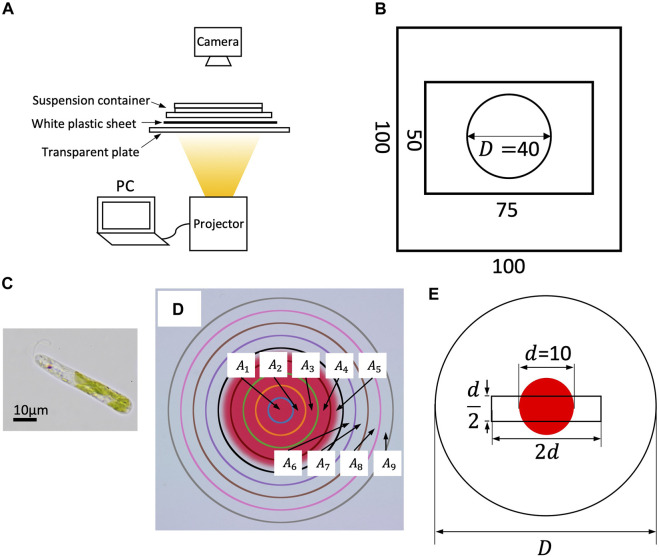
**(A)** Experimental setup. **(B)** Dimensions of suspension container. A silicone plate with 0.5 mm thickness is sandwiched between two rectangular glass plates. The suspension is put inside the circle (cylinder). **(C)** An image of *E. gracilis*. **(D)** Sub-regions for cell counting. **(E)** The search region of orbit analysis (a *d*/2×2*d* rectangle).

The experimental setup is shown in Figure 1A. The suspension container was illuminated from below by a small DLP projector with LED light source (PicoCube X, Felicross); the light field was prepared by the projection pattern generated on a PC through a white plastic sheet to diffuse light.

A suspension container was prepared using a silicone plate of 0.5 mm thickness with a circular hole *D* = 40 mm in diameter, sandwiched between a 100 mm × 100 mm glass plate and a 50 mm × 75 mm glass plate to generate a cylinder (*D* = 40 mm in diameter and 0.5 mm in thickness; Figure 1B).

**FIGURE 2:**
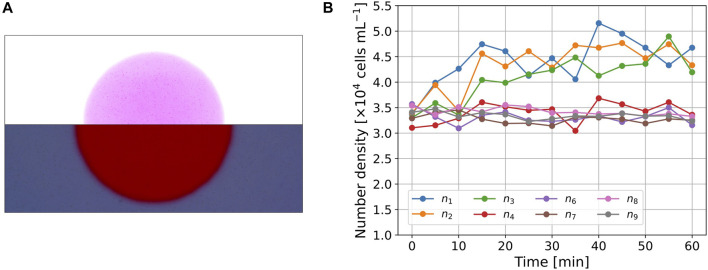
**(A)** Images of the cell distribution (*t* =30 min). The picture was divided into upper and lower parts, both of which were modified independently to visualize the cells for presentation purposes. **(B)** Time series of number density *n*_*k*_(*t*) in the subregions.

The suspensions were prepared as follows: *E. gracilis* (Figure 2C) was pre-cultured in Koren-Hutner medium for 2–4 weeks with continuous light illumination. The cells were then inoculated into 2 g L^−1^ HYPONeX solution with periodic light illumination (15 h bright light, 9 h dark). After 10–30 days of culture in HYPONeX aqueous solution, the suspensions were used for each experiment between the circadian time (CT) 8–12 h. The HYPONeX culture was diluted to 2.35 × 10^4^ cells mL^−1^ and sealed in a container. The volume fraction of the cell was *O* (10^–4^), where *O* (10^*a*^) means “order of magnitude of 10^*a*^”.

We performed two experiments (Experiments A and B).

In Experiment A, the temporal change in cell density under a heterogeneous light environment was quantitatively measured under a light field with two different intensity regions (Figure 1D).

Two patterns were prepared. Pattern 1 had a red filled circle of diameter *D*; the color was defined by RGB = (255, 64, 64) (denoted as ‘red’ hereafter). Pattern 2 had a smaller red filled circle of diameter *d* = 10 mm, and the outside color was defined by RGB = (255, 255, 255) (‘white’). Because *E. gracilis* does not recognize red color, the color difference between them consists of green and blue elements; the red element was included for image analysis. The photoflux density (PFD) of the projected colors was 18.7 *μ*mol m^−2^ s^−1^ (838 LUX) for red and 53.2 *μ*mol m^−2^ s^−1^ (3252 LUX) for white, based on an average of 240 measurements with an exposure time of 500 *μ*s using a spectrometer (MK350S Premium, UPRtek). Because the container diameter is equal to the diameter of Pattern 1, it provides a homogeneous light environment in the container, whereas Pattern 2 provides a heterogeneous light environment. Because the red region has an amount of light intensity, the light intensity difference is not analogous to the step-up light condition (Tsang et al., 2018; Ozasa et al., 2019).

The experimental system was kept motionless in dark conditions for 1 h before illumination with Pattern 1 for 10 min (State A) and then with Pattern 2 for 60 min. During illumination, the cells in the entire region were photographed every 5 min using a digital camera (Nikon Z7 II) with a macro lens (Nikkor Z 50 mm f/1.8 S). The focal depth was approximately 0.375 mm.

The whole region was divided to nine sub-regions *A*_*k*_ (*k* = 1, … , 9) defined by

$$A_{k}=\left\{\left(x,y\right)|\left(r_{k-1}^{2}\leq x^{2}+y^{2}<r_{k}^{2}\right),\;r_{k}=\frac{2k}{9}\frac{d}{2},1\leq k\leq 9\right\}$$ (1)

(Figure 1D). Here, *A*_5_ includes the boundary of the circle, where the cell number was not counted because of non-uniform contrast. The largest area *A*_9_ has diameter 2*d*, half that of *D*. Therefore, we assumed that the counted cell number was not affected by the sidewall. The cell number in the sub-region *A*_*k*_ was counted by image analysis using ImageJ. The image was converted to an 8-bit black-and-white image and binarized with a threshold to count the number. The threshold values for the red circle and that for the outside region were adjusted such that the cell number densities at *t* = 0 were at a same level. The number density in subregion *A*_*k*_ at time *t* is denoted by *n*_*k*_(*t*).

In Experiment B, cell orbits were recorded. The experimental procedure was the same as that in Experiment A, until State A. After State A, in a homogeneous light environment, the culture was illuminated with Pattern 1 for 30 min before recording the orbit for 120 s at a rate of 25 fps. In a heterogeneous light environment, the same experiment was performed using Pattern 2. Each experiment was performed twice in each of the two environments.

We selected all the moving cells at *t* = 0 inside the search region, a *d*/2 × 2*d* rectangle, at the center of the container in both environments. The rectangle contains both red and white regions of Pattern 2; the areas for both colors are approximately equivalent (Figure 1E). All cells inside the search region were tracked at 1/25 s intervals using a computer digital tracking software (DIPP-Motion, DITECT) in both environments.

MSD of the orbit ***X*** (*t*) was defined as.

$$MSD\left(t\right)=\left\langle \Delta X{\left(t\right)}^{2}\right\rangle ,$$ (2)

$$\Delta X{\left(t\right)}^{2}=|X\left(t\right)-X\left(0\right){|}^{2},$$ (3)

where ⟨⋅⟩ denotes the ensemble average and Δ*X*(*t*)^2^ is the squared displacement.

To exclude short-time motion such as helical orbit and measuring noises, and obtain an orbit with a long timescale, a moving average was performed. We use the term ‘*T* − sec averaged orbit’ for the orbit data after the moving average for the time interval of *T* s, $\int_{t-T/2}^{t+T/2}X\left(t^{\prime }\right)dt^{\prime }$ .

## 3 Results

### 3.1 Cell density in a heterogeneous environment

Figure 2A shows a snapshot of the picture (*t* = 30 min) in Experiment A. The images were divided into upper and lower parts, and their brightness and contrast were independently modified to clearly show the cells in both regions. The cell number density inside the circle was larger than that outside the circle. The cells near the boundary could not be clearly detected owing to spatial changes in light intensity. Thus, we omitted the cell number data for *A*_5_, *n*_5_(*t*), which contains the boundary. The initial cell number density was above that of the suspension because the cells accumulate due to negative phototaxis.

For quantitative analysis, *n*_*k*_(*t*) was measured, as shown in Figure 2B. The values of *n*_*k*_(*t*) (1 ≤ *k* ≤ 3), which correspond to the region inside the circle, show an increase in the initial interval 0 ≤ *t* ≤ 30 min and maintain larger values in the interval 30 < *t* < 60 min. The values of *n*_4_(*t*) are also the cell number densities inside the circle, but the initial value, *n*_4_(0), is smaller than the other values of *n*_*k*_(0) (*k* ≠ 4). The values of *n*_4_(*t*) showed a slight increase on average, but the trend was clearly smaller than the values of *n*_*k*_(*t*) (1 ≤ *k* ≤ 3). The cell density for the outer region, *n*_*k*_(*t*) (6 ≤ *k* ≤ 9), maintained similar values throughout the entire time interval 0 ≤ *t* ≤ 60 min, and the trend throughout the measurement period was almost constant or slightly decreased.

In Experiment A, we conclude that the cells of *E. gracilis* moves to red region in this setup and a balance between photomovement due to spatial light difference and the diffusion achieved after *t* = 30 min.

### 3.2 Cell orbit analysis

#### 3.2.1 Orbital characteristics in homogeneous and heterogeneous light environments

Among all the moving cells in the search region, some disappeared or adhered to the upper glass before reaching the prescribed measured time (120 s). The tracked individuals were categorized as follows: (I) swimming individuals that went out of the focal plane or were adhered before the measurement time ended; (II) swimming individuals that stayed inside the focal plane until the measurement time. In a homogeneous environment, there were 79 orbits ((I) 14, (II) 65). In a heterogeneous environment, there were 81 orbits ((I) 30, (II) 51). Because the focus depth was smaller than the depth of the container, the motion of the disappeared cells was more three-dimensional. To test whether the difference in motion is related to the environment, we performed Fisher’s exact test for the cell numbers in categories (I) and (II) with the null hypothesis that “The cell motions are independent of the environments,” which was rejected at the 1% level (*p* = 0.00779), implying that the heterogeneous environment is related to the vertical cell motion, although the gradient of the light intensity is horizontal. In the following analysis, we focus on the orbits in category (II) to examine horizontal cell motion in different environments.

#### 3.2.2 Orbit and speed distribution

**FIGURE 3:**
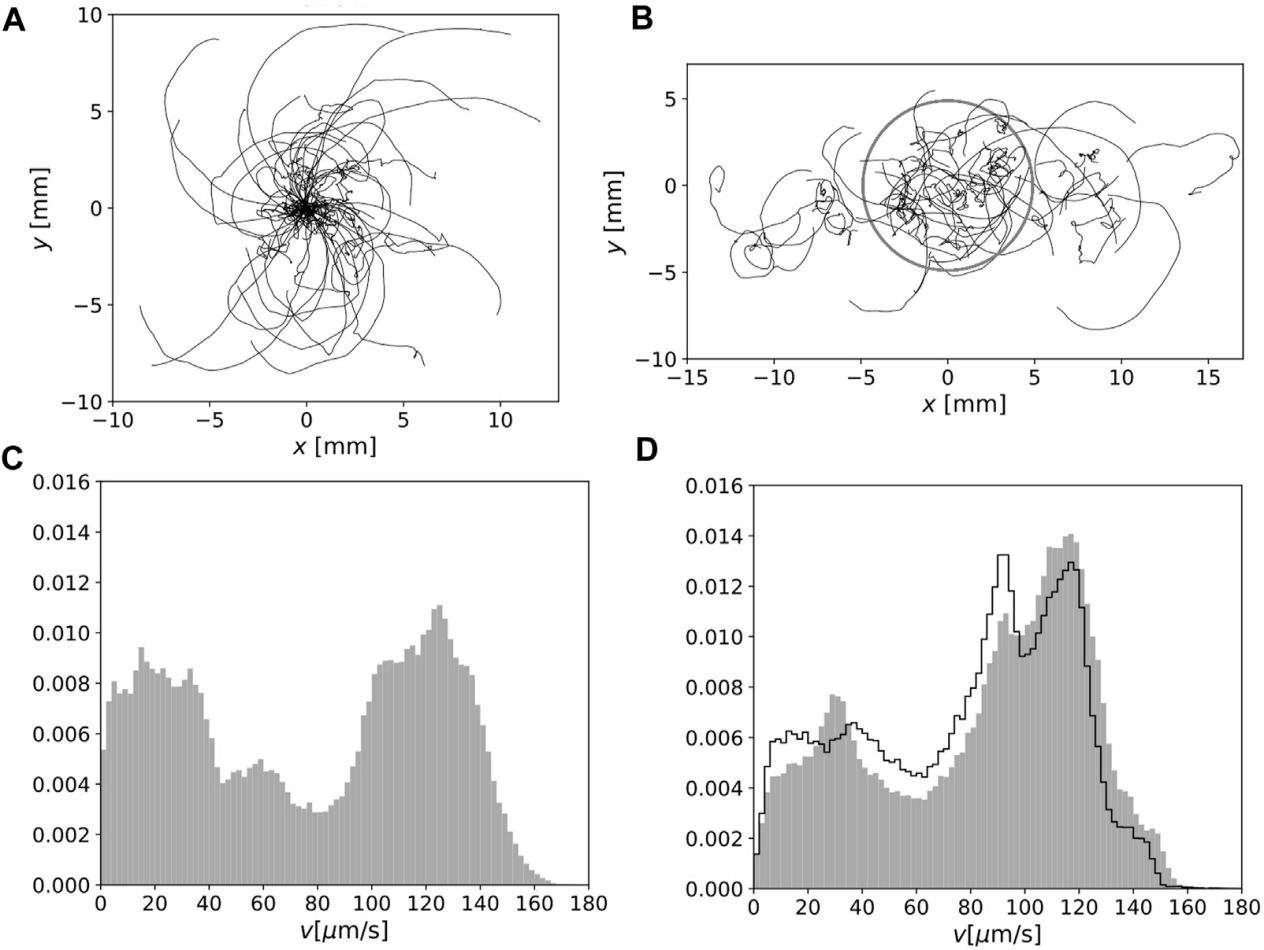
**(A)** Cell motion for 120 s in homogeneous environment. The initial positions were set at the origin. **(B)** As in **(A)** but under heterogeneous light conditions in the laboratory frame. The boundary of the red region is indicated by a grey circle. **(C)** Normalized speed distribution for 1 s-averaged orbits in a homogeneous environment. **(D)** As in **(C)** but in a heterogeneous environment. The normalized distribution of the speed of the orbits inside the red region is also indicated by the solid lines.

The orbits of *E. gracilis* obtained in Experiment B are shown in Figure 3A (homogeneous environment) and Figure 3B (heterogeneous environment). In Figure 3A, all orbits’ initial position was shifted to the origin because of homogeneity. In this figure, the orbits of the spatial scale from *O* (10^3^) to *O* (10^4^) *μ*m are visible; many orbits consist of straight or curved parts and occasional direction changes, although some orbits change direction more randomly. In Figure 3B, the orbits in a heterogeneous environment are shown in the laboratory frame. The grey circle indicates the boundary of the red region.

The distributions of the local speed for the 1 s-averaged orbit are shown in Figure 3C (homogeneous environment) and Figure 3D (heterogeneous environment). The faster swimming fraction in a heterogeneous environment was enhanced. To confirm this difference, we sampled the orbits inside the red region in a heterogeneous environment (Figure 3D, solid lines); however, the distribution was clearly different from the distribution in Figure 3C.

### 3.3 Long-time behavior: Analysis for moving-averaged orbit

#### 3.3.1 Joint distribution of speed and curvature radius

**FIGURE 4:**
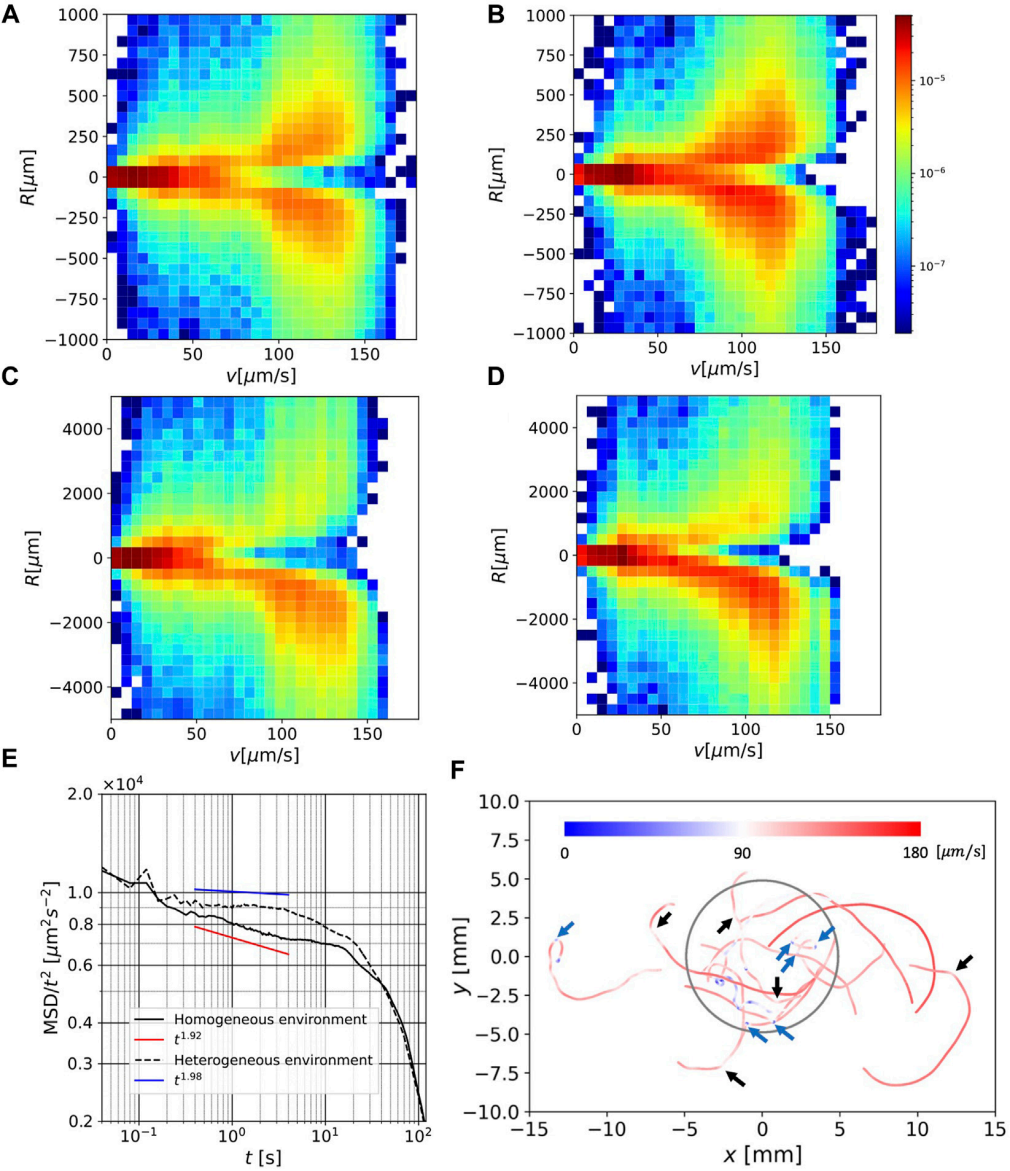
**(A)** Joint distribution of speed *v* and curvature radius *R* for 1 s-average orbit in a homogeneous environment. **(B)** As in **(A)** but in a heterogeneous environment.**(C)** As in **(A)**, but for 10 s-average orbit. **(D)** As in **(C)** but in a heterogeneous environment. **(E)** MSDs for all orbits under homogeneous and heterogeneous conditions. **(F)** Selected 5 s-average orbits in a heterogeneous environment, colored by local speed. The blue parts indicate speeds less than 90 *μ*m/s and the red parts indicate speeds larger than 90 *μ*m/s.

Figure 4A and Figure 4B show joint distributions of speed *v* and curvature radius *R* for the 1 s-averaged orbits in homogeneous and heterogeneous environments, respectively. The distribution is symmetric with respect to the *v*-axis, suggesting that the orbit curve direction has no bias at this timescale. Although the density is biased to the faster speed region for heterogeneous environment, the values of *R* that give the peak of distribution for fixed *v* did not show significant differences in either environment.

Figures 4C,D each show similar joint distributions in Figures 4A,B, but for the 10 s-averaged orbits. The range of *R* in these figures extends to values much larger than those in Figures 4A,B. We note that the curvature radius is defined by local information (swimming speed and acceleration) alone, but the curvature radius of the moving time-averaged orbit contains large-scale shape information.

In this case, the distribution is clearly biased toward negative *R*, suggesting that the (large-scale) orbit curve direction is predominantly clockwise. The peak value at *v* = 120 *μ*m is given in *R* ≃ 1000 *μ*m, which is of the same order as the curvature radius of the orbits in Figures 3A,B. Interestingly, the value of *R* that gives the peak for a fixed *v*, does not show a significant difference between the two environments. However, in a heterogeneous environment, the speed distribution is shifted to the faster region, and thus, the orbit in the clockwise direction is predominant (*cf.* Figures 3A,B).

#### 3.3.2 MSD

Figure 4E shows the MSDs in the homogeneous and heterogeneous environments. These were divided by *t* ^2^ to clearly show the difference from the ballistic orbit (MSD ∼ *t* ^2^). In the long range of the timescale, 0.2 < *t* < 40 s, the values of MSD in a heterogeneous environment are greater than those in a homogeneous environment, which is consistent with the speed distribution in a heterogeneous environment having a greater peak in the fast regime. The differences in the MSD curves outside the range nearly overlapped. For orbits in a homogeneous environment, the MSD shows a power-law region in the range *t* < 4. A fit line using the data in 0.4 < *t* < 4 with an exponent of 1.92 was drawn. A power-law region observed in the heterogeneous environment in 0.4 < *t* < 4 was close to ballistic, and a fit line using the same data range with an exponent of 1.98 was drawn. In both environments, the MSD deviates from *t* ^2^, and the exponent seems to decrease over a large timescale (*t* > 10).

#### 3.3.3 Orbit shape and speed

Figure 4F shows the 5 s-averaged orbits in a heterogeneous environment, selected on the condition $X{\left(120\right)}^{\frac{1}{2}}>5\times 10^{3}\mu$ m. The average time was chosen so that the difference between MSDs in the two environments was most distinct (c.f. Figure 4E), and the critical displacement length was determined by approximating MSD at *t* = 120 s. The orbit was colored with the local speed, red (*v* > *v*_*c*_) and blue (*v* < *v*_*c*_), where *v*_*c*_ = 90 *μ*m/s.

Most of the large-scale curves of the radius *O* (10^3^) − *O* (10^4^) *μ*m are colored red, which indicates moving with a large curvature radius is characterized by a fast-swimming speed. This is consistent with the fact that a fast swimming speed is related to a large curvature radius (Figure 4D). This speed persistence is also consistent with the MSD, which exhibits a ballistic region.

Another interesting feature is the occasional quick change in swimming direction at a slow speed. Several significant directional changes on this spatial scale are indicated by blue arrows, and mild but apparent directional changes are indicated by black arrows. Although the light intensity changes at the boundary of the red region in a heterogeneous environment, no distinct orbital characteristics are observed near the boundary (Figure 3B; Figure 4F).

## 4 Discussion: How *E. gracilis* swims for longer-time scale in a heterogeneous environment

For *E. gracilis*, the time to swim for the circle diameter is approximately 10^2^ s, which is longer than the adaptation timescale for the step-down photophobic responses, 20 s (Matsunaga et al., 1998), and of the same order of the relaxation time for the polygonal orbit after a sudden change in light intensity, ∼120 s (Tsang et al., 2018). Considering that a swimming orbit can cross a circle with a shorter path, our experimental setup allowed microorganisms to experience successive changes in the environment with the same order of adaptation time. In this sense, this experimental setup provides a “diorama environment”, an artificial condition to find potential adaptability in *E. gracilis*. As shown in Figure 3D, heterogeneous light condition is not simple superposition of two homogeneous illuminations with different light intensities.

Our orbit analysis suggests that orbits in a heterogeneous environment tend to be off the focus depth (Section 3.2.1), in other words, the cell motion in a heterogeneous environment is more three dimensional than that in homogeneous environment. The analysis of all individuals in the search region suggests that the speed distribution in a heterogeneous environment is different from that in a homogeneous environment (Figure 3).

The curvature radius of the orbit *R*, is strongly related to the local speed *v* for a long timescale (both the 1 s and 10 s timescales; Figures 4A–D), which is longer than the periods during helical trajectories by Rossi et al. (2017). This is consistent with the fact that single-flagellar swimming of *E. gracilis* restricts swimming behavior. The steering system of *E. gracilis* adapted to various environments, which is an important element in constructing the adaptation algorithm, should be sufficiently simple.

In view of the algorithm, the result that the bias of the swimming direction depends on the timescale is interesting. These results lead us to hypothesize that *E. gracilis* has a long timescale steering algorithm in which the curvature radius of orbit determined by speed. For swimming, the steering direction can be both clockwise and counterclockwise for a short timescale (1 s) but clockwise alone for a long timescale (10 s). These swimming behaviors appear to be the same in both environments. Non-etheless, the environmental difference leads to a difference in the 1 s scale orbit characteristics in the MSD.

In the long timescales (0.2 < *t* < 40), the MSD in a heterogeneous environment takes larger values than in a homogeneous environment (Figure 4E). In a homogeneous environment, the orbit had a power-law range, where it was close to ballistic but contained small randomness (the exponent was approximately 1.92), which is consistent with that of a previous study (Ogawa et al. (2017)). In a heterogeneous environment, the orbit had a ballistic range (the exponent was close to 1.98). These results allowed us to propose a hypothesis that the cells may use a strategy to search for a more favorable environment (darker region) by enhancing MSD in a heterogeneous environment. However, this remains an open question. For very long timescales (*t* > 40), the MSD deviates from (semi-) ballistic behavior, and the behavior is more diffusive. These differences may reflect the different behaviors of *E. gracilis* in different environments.

Figure 4F shows that the speed in straight or large-scale (*O* (10^3^)*μ*m) curved parts in orbit is fast, whereas the speed during direction change between these parts is slow. Such switching strategy reminds us of the ‘run-and-tumble’ algorithm for *E. coli* (Berg, 1993), where chemotaxis is achieved by switching between straight swimming and random direction change during an increasing or decreasing favorable chemical concentration, respectively. In our environment, however, the light gradient occurred in a narrow region, and we did not observe characteristic behavior near the boundary (Figure 3B; Figure 4F).

The results suggest that long-term and large-scale behavior is different from short-term and small-scale behavior, which can be related to the sudden change in light intensity. The photomovement of individuals under a spatial light gradient has been investigated by Ogawa et al. (2017); however, long-time orbits in heterogeneous media have not been analyzed.

For instance, a transition from ballistic to diffusive behavior observed in some theoretical models (Solon et al., 2015; Zaburdaev et al., 2015; Marchetti et al., 2016) may provide additional information for the algorithm for *E. gracilis* motion in a heterogeneous environment, especially for the spatial scale of heterogeneity. Further investigation for this is warranted.

## 5 Conclusion

We analyzed the cell motion of *E. gracilis* in homogeneous and heterogeneous light environments, where a heterogeneous light environment was prepared by a red circle region. The spatiotemporal cell number density showed that the cells moved into the red region. In the equilibrium state, in which the cell motion due to light difference balances with the diffusive motion, we tracked the cell motion to reveal that the speed distribution for the 1 s-averaged cell orbits in both environments showed a difference in that the high-speed fraction becomes greater in a heterogeneous environment. The joint histograms for the speed and curvature radius of the orbits in a short timescale (1 s-averaged) and long timescale (10 s-averaged) orbits were compared. The orbits in the short timescale provide a symmetric distribution of the curvature radius at a given speed in all speed regimes in both light environments. In the long timescale, the distribution of the curvature radius is strongly biased in the clockwise direction in the faster speed regime in both light environments. Power law exponent of MSD in a homogeneous environment was observed in the timescales *t* ∼ 1 s and agreed with previous measurements by Ogawa et al. (2017). The MSD in the heterogeneous environment showed an almost ballistic regime at the same timescale. At this timescale, the MSD in a heterogeneous environment is larger than that in a homogeneous light environment.

Here, we demonstrated quantitative differences in long-time swimming behavior in a heterogeneous environment. The detailed algorithm for searching and moving to the favorite (red) region may be determined in the future and compared with known algorithms, such as the ‘run-and-tumble’ mechanism.

## Data Availability

The raw data supporting the conclusion of this article will be made available by the authors, without undue reservation.

## References

[B1] Berg H. C. (1993). Random walks in Biology paperback. Princeton University Press.

[B2] Diehn B. (1973). Phototaxis and sensory transduction in Euglena. Science 181, 1009–1015. 10.1126/science.181.4104.1009 4199225

[B3] Giometto A. Altermatt F. Maritan A. Stocker R. Rinaldo A. (2015). Generalized receptor law governs phototaxis in the phytoplankton Euglena gracilis. Proc. Natl. Acad. Sci. 112, 7045–7050. 10.1073/pnas.1422922112 25964338 PMC4460502

[B4] Giuliani N. Rossi M. Noselli G. DeSimone A. (2021). How Euglena gracilis swims: Flow field reconstruction and analysis. Phys. Rev. E 103, 023102. 10.1103/PhysRevE.103.023102 33736112

[B5] Iseki M. Matsunaga S. Murakami A. Ohno K. Shiga K. Yoshida K. (2002). A blue-light-activated adenylyl cyclase mediates photoavoidance in Euglena gracilis. Nature 415, 1047–1051. 10.1038/4151047a 11875575

[B6] Kato S. Shinomura T. (2019). Eyespot structure and its function in photobehaviors of phytoflagellate. PLANT Morphol. 31, 3–9. 10.5685/plmorphol.31.3

[B7] Marchetti M. C. Fily Y. Henkes S. Patch A. Yllanes D. (2016). Minimal model of active colloids highlights the role of mechanical interactions in controlling the emergent behavior of active matter. Curr. Opin. Colloid and Interface Sci. 21, 34–43. 10.1016/j.cocis.2016.01.003

[B8] Matsunaga S. Hori T. Takahashi T. Kubota M. Watanabe M. Okamoto K. (1998). Discovery of signaling effect of UV-B/C light in the extended UV-A/blue-type action spectra for step-down and step-up photophobic responses in the unicellular flagellate alga Euglena gracilis. Protoplasma 201, 45–52. 10.1007/bf01280710

[B9] Noselli G. Beran A. Arroyo M. DeSimone A. (2019). Swimming *Euglena* respond to confinement with a behavioral change enabling effective crawling. Nat. Phys. 15, 496–502. 10.1038/s41567-019-0425-8 31110555 PMC6522345

[B10] Ogawa T. Izumi S. Iima M. (2017). Statistics and stochastic models of an individual motion of photosensitive alga Euglena gracilis. J. Phys. Soc. Jpn. 86, 074401. 10.7566/jpsj.86.074401

[B11] Ogawa T. Shoji E. Suematsu N. J. Nishimori H. Izumi S. Awazu A. (2016). The flux of Euglena gracilis cells depends on the gradient of light intensity. PLOS ONE 11, e0168114. 10.1371/journal.pone.0168114 28033336 PMC5199022

[B12] Ozasa K. Won J. Song S. Shinomura T. Maeda M. (2019). Phototaxis and photo-shock responses of Euglena gracilis under gravitaxis. Algal Res. 41, 101563. 10.1016/j.algal.2019.101563

[B13] Rossi M. Cicconofri G. Beran A. Noselli G. DeSimone A. (2017). Kinematics of flagellar swimming in Euglena gracilis: Helical trajectories and flagellar shapes. Proc. Natl. Acad. Sci. 114, 13085–13090. 10.1073/pnas.1708064114 29180429 PMC5740643

[B14] Solon A. P. Cates M. E. Tailleur J. (2015). Active brownian particles and run-and-tumble particles: A comparative study. Eur. Phys. J. Special Top. 224, 1231–1262. 10.1140/epjst/e2015-02457-0

[B15] Tsang A. C. H. Lam A. T. Riedel-Kruse I. H. (2018). Polygonal motion and adaptable phototaxis via flagellar beat switching in the microswimmer Euglena gracilis. Nat. Phys. 14, 1216–1222. 10.1038/s41567-018-0277-7

[B16] Zaburdaev V. Denisov S. Klafter J. (2015). Lévy walks. Rev. Mod. Phys. 87, 483–530. 10.1103/revmodphys.87.483

